# Disparities in access to preventive health care services among insured children in a cross sectional study

**DOI:** 10.1097/MD.0000000000004262

**Published:** 2016-07-18

**Authors:** Christian King

**Affiliations:** Department of Nutrition and Health Sciences, University of Nebraska–Lincoln, Lincoln, Nebraska.

**Keywords:** access to care, child health, health insurance, preventive care, social inequality, underinsurance

## Abstract

Children with insurance have better access to care and health outcomes if their parents also have insurance. However, little is known about whether the type of parental insurance matters. This study attempts to determine whether the type of parental insurance affects the access to health care services of children.

I used data from the 2009–2013 Medical Expenditure Panel Survey and estimated multivariate logistic regressions (N = 26,152). I estimated how family insurance coverage affects the probability that children have a usual source of care, well-child visits in the past year, unmet medical and prescription needs, less than 1 dental visit per year, and unmet dental needs.

Children in families with mixed insurance (child publicly insured and parent privately insured) were less likely to have a well-child visit than children in privately insured families (odds ratio = 0.86, 95% confidence interval 0.76–0.98). When restricting the sample to publicly insured children, children with privately insured parents were less likely to have a well-child visit (odds ratio = 0.82, 95% confidence interval 0.73–0.92), less likely to have a usual source of care (odds ratio = 0.79, 95% confidence interval 0.67–0.94), and more likely to have unmet dental needs (odds ratio = 1.68, 95% confidence interval 1.10–2.58).

Children in families with mixed insurance tend to fare poorly compared to children in publicly insured families. This may indicate that children in these families may be underinsured. Expanding parental eligibility for public insurance or subsidizing private insurance for children would potentially improve their access to preventive care.

## 1 Introduction

The ability to access preventive health care services is an important factor contributing to the health of children and their development.^[1]^ The American Academy of Pediatrics guidelines recommend regular well-child visits for young infants and annual visits for children ages 3 and older.^[2]^ Well-child visits reduce hospitalizations,^[3]^ reduce emergency department use,^[4,5]^ and improve child health.^[6,7]^ In addition, having a usual source of care leads to higher use of preventive care,^[8,9]^ lower use of emergency department,^[10,11]^ and a reduction in unmet medical and prescription needs.^[12]^

Child health insurance status is a strong predictor of access to preventive health care services. Uninsured children face barriers in accessing health care services,^[13,14]^ are less likely to have a usual source of care,^[15,16]^ leading to poorer health outcomes and health complications in the future.^[17]^ Recent expansions in public insurance in the United States (through Medicaid and the Children's Health Insurance Program, CHIP) over the past decade have substantially increased the health insurance coverage of children, leading to better access to health care services.^[18,19]^ At the same time, the cost of dependent coverage under employed-sponsored insurance has increased over time, leading more parents to forgo enrolling their children under their private insurance and enrolling them in public insurance instead.^[20,21]^ A report by the Government Accountability Office estimated that between 2005 and 2007 about 4% of children (or about 3 million) in the United States had public insurance while their parent were privately insured.^[22]^ This number increased to 4.3 million in a 2011 report analyzing the 2009 American Community Survey (ACS).^[23]^

There is evidence of disparities in access to care among insured children.^[24]^ Some of these disparities are a result of underinsurance when children while continuously insured, are inadequately covered.^[25]^ This inadequate coverage can take the form of cost sharing that are too high, a limited level of benefits, or inadequate coverage of needed services. While there is evidence that individual coverage leads to different outcomes, how family coverage affects children is not well understood. It remains largely unknown whether children in families covered under different sources of insurance experience different access to preventive health care services than children in families under a single source of insurance coverage (private or public).

Using data from the 2009–2013 Medical Expenditures Panel Survey (MEPS), this study explored the association between the type of family health insurance and access to health care services for children. The analysis compared among 3 different groups of insured children: children in families under private insurance, children in families under public insurance, and publicly insured children in families with privately insured parent(s).

Some have argued that health disparities and underinsurance in the United States and other countries are a result of social inequalities.^[26]^ In other words, families with higher socioeconomic status have better health care access and health insurance coverage, which leads to better health outcomes. Understanding these health insurance dynamics is important for policy and reduce some of these social inequalities. Should more families and children be covered by public insurance or should they receive subsidies for private insurance if parents are unable to cover dependents under their employer-sponsored insurance? One study argues that Medicaid expansions alone might not improve access to care.^[27]^ In other words, public insurance expansions may not be an optimal one size-fits-all solution.

Underinsurance and health disparities also contribute to the high costs of health care and its inefficiencies. People who are inadequately covered do not use preventive services as much as they need.^[24]^ This may lead them to use emergency services when it is unavoidable and too late for conditions that were preventable in the first place, which may make them financially vulnerable. In addition, hospitals also become financially vulnerable as they have to provide care that is often uncompensated.^[28]^ As a result, other patients may bear these costs.

## 2 Methods

### 2.1 Data

This study used the MEPS from the Agency for Healthcare Research and Quality, a yearly nationally representative survey of noninstitutionalized US households. The survey asks households about the health care utilization, spending, and insurance pattern of each member during the previous year. This analysis used the Household Component, which collects data for each person in the household on demographic characteristics, health conditions, and health status among others. Each household is present for 2 years in the sample. This analyses pools data from 2009 to 2013. The research was carried out in compliance with the Helsinki Declaration. Since this study uses national data that are publicly available, I did not need to obtain individual consent.

To examine the association between family health insurance coverage and the access to health care services of children, I restricted the sample to households with children (n = 53,555). Households with heads above 65 were dropped because these individuals are eligible for Medicare. Households with an uninsured member were dropped to avoid having the association of uninsurance confounding the results. A small number of families with children had multiple sources of health insurance coverage and were dropped. The analytical sample includes 26,152 children.

### 2.2 Measures

I used 5 outcome variables that have been shown to be association with child insurance and access to health care services^[20,29–31]^ which included: having a usual source of care, having a well-child visit in the past year, having unmet medical or prescription needs, having less than 1 dental visit per year, and having unmet dental needs.

Mutually exclusive binary variables are constructed to distinguish between 3 different familial health insurance coverage. First, families that have all members covered under private insurance. Second, families that are covered under public insurance. Third, families with members under different insurance coverage. These families either have a privately insured parent with a publicly insured child or a publicly insured parent with a privately insured child. Given that the latter is less common, these families with mixed insurance coverage mainly have parents under private insurance and children under public insurance.

The analysis controlled for several characteristics that may confound the association between family insurance and health outcomes, which includes gender and age of the child, race/ethnicity(White, Black, Asian, Hispanic, and other race), highest education achieved by either parent (less than high school, high school, some college, and college graduate),the employment status of the head of the household and the spouse, the number of children in the household, and income categories (<100% Federal Poverty Level [FPL], between 101% and 124% FPL, between 125% and 199% FPL, between 200% and 399% FPL, and more than 400% FPL). Lastly, dummy variables for survey years are included.

### 2.3 Analysis

First, the analysis usedunivariate logistic regression to show the unadjusted association between the different types of health insurance on children's access to health care services. Second, the analysis used multivariate logistic regression adjusting for all the variables described to examine these associations. Two different models are shown. The first one compared among all insured children. Second, because children in families under private insurance are likely to have better health and be better off than other children, additional models are estimated restricting the sample to children in families with public or mixed insurance to compare these children directly. Most of the variables used in the study did not have missing values. All the analyses were conducted using Stata 13 (StataCorp LP, College Station, TX). To account for the survey sampling design, the analysis used the SVY command in Stata.

## 3 Results

This study has a sample of 26,152 children. Table 1 presents summary statistics by family insurance coverage. About 56.4% of children in this sample lived in privately insured families. Over about a quarter (27.2%) of children in this sample lived in publicly insured families, and the remaining (16.4%) children lived in families with mixed insurance coverage (ie, parent privately covered and child publicly covered).

**Table 1:**
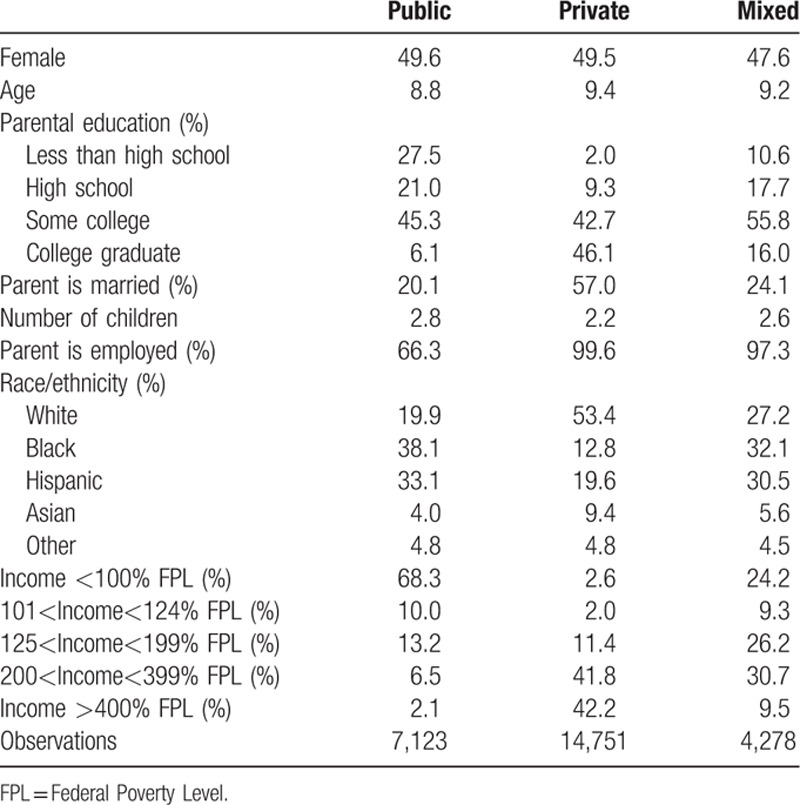
Characteristics of insured children by insurance status MEPS 2009–2013.

There were also large disparities in demographic and socioeconomic characteristics of children of different health insurance coverage. Around 88.8% of privately insured children had a parent who completed some college or was a college graduate, compared to about 71.8% for mixed insured children and about 51.4% for publicly insured children. African-American and Hispanic children were substantially more likely to be in families with public insurance or mixed insurance (71.2% and 62.6%) than private insurance (32.4%). Privately insured children were substantially more likely to live in families with incomes of more than 200% of the federal poverty level.

Table 2 presents estimates for the univariate logistic regressions reporting odds ratio. The reference group represents children under private insurance. The unadjusted models show that both children with mixed insurance and publicly insured children are less likely to have a well-child visit, less likely to have usual source of care and are more likely to have less than yearly dental visits compared to privately insured children.

**Table 2:**
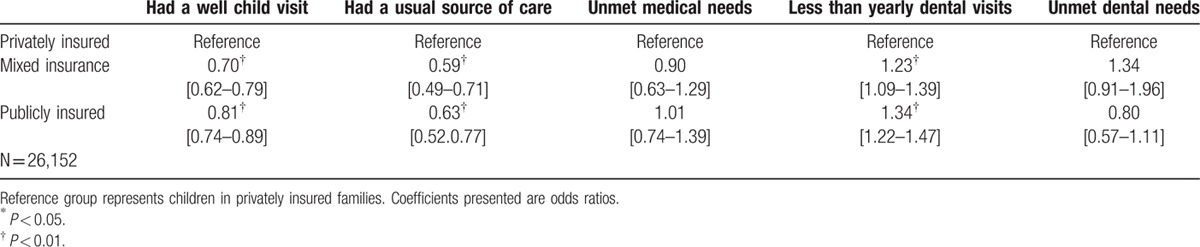
Univariate logistic regressions showing the association between family insurance coverage on health care access among insured children. MEPS 2009–2013.

After adjusting for potential confounders (Table 3), children in mixed insured families are less likely (odds ratio = 0.85, 95% confidence interval 0.76–0.98) to have a well-child visit in the past year. The remaining table shows that odds ratio are not statistically significant, which means that most of the differences observed can be explained by socioeconomic status and other potential confounders.

**Table 3:**
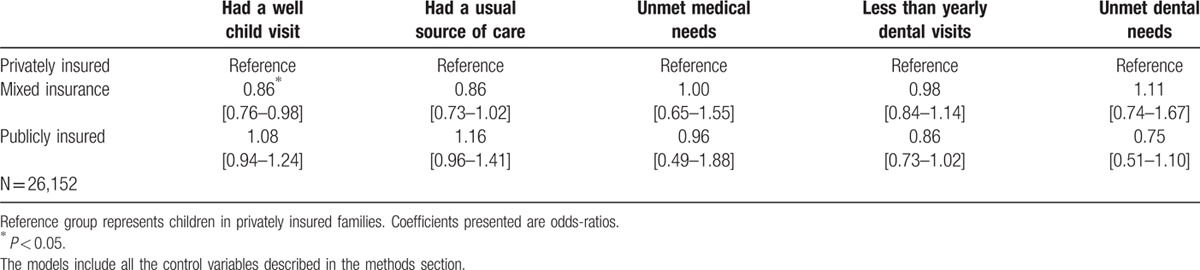
Multivariate logistic regressions showing the association between family insurance coverage on health care access among insured children. MEPS 2009–2013.

Comparing across all insured children might not be optimal. Privately insured children may not be an adequate comparison group, as they tend to be healthier because their families may have greater resources. Table 4 presents the same multivariate estimates excluding privately insured children from the sample and comparing directly between children under public insurance to children in families with mixed insurance.

**Table 4:**
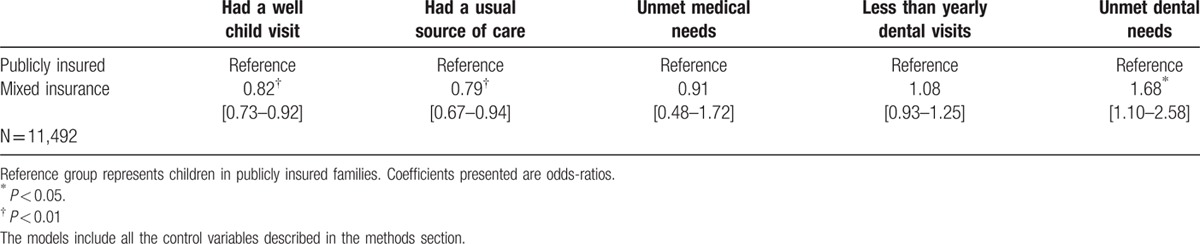
Multivariate logistic regressions showing the association between family insurance coverage on health care access among insured children. MEPS 2009–2013.

Compared to children in publicly insured families, children in mixed insured families are less likely to have a well-child visit in the past year (odds ratio = 0.82, 95% confidence interval 0.73–0.92). They are also less likely to have a usual source of care (odds ratio = 0.79, 95% confidence interval and more likely to have unmet dental needs (odds ratio = 0.168, 95% confidence interval 1.10–2.58). These differences are statistically significant and indicate that children under mixed insurance do not have the same access to preventive care than children in publicly insured families.

## 4 Discussion

Public insurance remains an important source of coverage for children, especially those from lower-income families who cannot afford private insurance. In 2014, close to 43% of children in the United States under the age of 19 were publicly insured.^[32]^ Previous studies have shown that providing insurance to previously uninsured children, often through Medicaid or CHIP expansions, substantially improves the health and access to care of these children.^[27]^ However, the type of insurance the parents are under also affects the access to health care services of children.

The findings of this study are important because they compare among insured children. This study showed that uniform family insurance coverage provides better access to health care services to children. Children who were in families with mixed insurance were less likely to utilize preventive care than children in publicly insured families. It is likely that families with mixed insurance are underinsured, which could explain the poorer outcomes of children in these families. Although underinsured families have continuous health insurance, the inadequacy of their coverage leads to suboptimal health care access and quality of care received.^[24]^ Underinsurance can be even more prevalent than uninsurance. One study shows that in 2007, the number of underinsured children in the United States (14.1 million) was even higher than the number of children without insurance (11 million).^[24]^ Another study estimated that in the United States, about one third of low-income adults were underinsured.^[33]^ Often, policy discussions focus on uninsured populations and overlook the underinsured.

While the study examined the context of the United States, the study is also relevant in the international and global contexts. The issue of underinsurance and health is complex and has several determinants. Leischik et al^[34]^ argue that social inequalities are some of the most important determinants of health. Those with higher socioeconomic status tend to have better access to health care services and better health outcomes. However, among developed countries, those with the highest level of health and life expectancy are not the richest ones, but the ones with the smallest social and income disparities and inequalities.^[34–36]^ On the other hand, the issue of health has some nuances and has different dimensions in different countries. For example, obesity tends to affect higher educated groups in developing countries, while it is more prevalent in lower educated groups in developed countries.^[37]^ Also, lower to middle income countries tend to have inadequate resources for health care, such as cancer care, which in some cases, requires large upfront investments for specialized types of cancer.^[38]^

Nevertheless, social inequalities appear to be some of the strongest determinants of health worldwide. In addition, family resources remains an important predictor of health and well-being for young people.^[39]^ The World Health Report from the World Health Organization calls for universal health coverage to improve health and well-being.^[26]^ One of the issues raised in the report is narrowing the gap between the poorest and richest quintiles in order to improve health outcomes in low and middle income countries.

Some empirical evidence shows that the underinsured face similar barriers in access to health care and have similar adverse health outcomes than uninsured individuals.^[24,40]^ Since social inequalities explain disparities in insurance coverage, the policy implications of this study are that reducing social inequalities would reduce uninsurance and underinsurance. This can be accomplished through either subsidizing parents so that they can enrol their children in their employer-sponsored insurance or having both parent and child enrolled in public insurance. Given that parents who receive employer-sponsored insurance typically do not qualify for public insurance, the income threshold to qualify would have to be revised. A cost-benefit analysis might be needed to determine whether subsidizing children to enrol in private insurance or whether increasing enrolments of parents from private to public insurance might be a more efficient and cost-effective alternative.

Providing adequate insurance coverage is one way to reduce social inequalities. The 2010 United States Affordable Care Act (ACA) has reduced underinsurance for young adults between the ages 19 and 25. Previously, this age group had high rates of uninsurance and underinsurance.^[41]^ The ACA has allowed these young adults to remain on their parents’ private plan. One study finds that this expansion of coverage also protected these previously uninsured or underinsured young adults from the disastrous financial consequences of a serious medical emergency.^[28]^ Before the reform, these young adults would use emergency services to treat preventable conditions that worsened as they were left untreated. As a result, the reform reduced the percentage of emergency care visits by uninsured young adults.^[28]^ In addition, the higher use of emergency care by uninsured young adults could have contributed to the high cost of health care since hospitals had to provide uncompensated care, which may mean that they needed to recoup their cost from other compensated services provided. The study finds that the provision reduced the amount of charity care provided.^[28]^

One strength of this study is the comparison among insured children. Uninsured children tend to have different characteristics than insured children that affect their health and access to care. The analysis used a few sensitivity checks to ensure that the findings were robust. Although accounting for income and health should account for some of these differences that may explain health and access to care disparities, some unobserved differences may remain. First, the analysis reestimated these models dropping all previously uninsured children since past uninsurance may be correlated with both health insurance coverage and access to health care services. Second, the analysis also dropped children in households in the highest income bracket (greater than 400% FPL) since they tend to be better off in both measurable and unmeasured characteristics. Third, the analysis also used propensity score matching to ensure that the comparison groups of insured children were as similar as possible. The results did not substantially change or deviate from the findings presented. The main limitation of the study is the cross-sectional nature of the data, which prevents establishing causal links. Despite these limitations, this study adds more understanding as to how the type of parental insurance affects the access to health care services of children.

## 5 Conclusion

Comparing among insured children shows that children under mixed insurance have similar access to health care services than privately insured children. However, privately insured children may not be an optimal comparison group. The results showed that among publicly insured children, children in families under mixed insurance face greater barriers in access to health care services. Children under mixed insurance were less likely to have a well-child visit in the past year, were less likely to have a usual source of care, and more likely to have unmet dental needs. The implications for policy are that expanding public insurance eligibility to parents or subsidizing private insurance for children would help families have uniform health insurance coverage, which would improve the access to health care services for children, especially publicly insured children. Further research is needed to understand the effect of mixed insurance on the health outcomes of these children.
